# How does the cut-off point for grip strength affect the prevalence of sarcopenia and associated factors? Findings from the ELSI-Brazil Study

**DOI:** 10.1590/0102-311XEN155624

**Published:** 2025-06-27

**Authors:** Sara Souza Lima, Roberta de Oliveira Máximo, Mariane Marques Luiz, Patrícia Silva Tofani, Letícia Coelho Silveira, Thales Batista de Souza, Thaís Barros Pereira da Silva, Valdete Regina Guandalini, Maria Fernanda Lima-Costa, Cesar Messias de Oliveira, Tiago da Silva Alexandre

**Affiliations:** 1 Programa de Pós-graduação em Gerontologia, Universidade Federal de São Carlos, São Carlos, Brasil.; 2 Programa de Pós-graduação em Fisioterapia, Universidade Federal de São Carlos, São Carlos, Brasil.; 3 Universidade Federal do Sergipe, Lagarto, Brasil.; 4 Programa de Pós-graduação em Nutrição e Saúde, Universidade Federal do Espírito Santo, Vitória, Brasil.; 5 Núcleo de Estudos em Saúde Pública e Envelhecimento, Fundação Oswaldo Cruz, Belo Horizonte, Brasil.; 6 Universidade Federal de Minas Gerais, Belo Horizonte, Brasil; 7 University College London, London, U. K.

**Keywords:** Sarcopenia, Grip Strength, Prevalence, Sarcopenia, Força da Mão, Prevalência, Sarcopenia, Fuerza de la Mano, Prevalencia

## Abstract

This study aimed to compare the prevalence of sarcopenia and associated factors with the definition of muscle weakness established using two different cut-off points for grip strength. We carried out a cross-sectional study with 7,065 participants (aged 50 or older) from the ELSI-Brazil Study. Sarcopenia was defined by the European Working Group on Sarcopenia in Older People (EWGSOP2) and two different cut-off points for grip strength to define weakness: < 27kg for men/< 16kg for women or < 36kg for men < 23kg for women. The prevalence of different sarcopenia status was estimated, and associations with sociodemographic, behavioral, and clinical factors were investigated using multinomial regression models. The mean age of participants was 61 years; 51.8% were women and 41.5% were at risk of malnutrition. By adopting the higher cut-off points (< 36kg/< 23kg) for the definition of weakness, the prevalence of probable sarcopenia and sarcopenia quadrupled (40.1% versus 10.6%; 5% versus 1.4%, respectively) and the prevalence of severe sarcopenia doubled (8.8 versus 3.9%). Nutritional status was associated with sarcopenia status, however the cut-off points < 36/< 23kg increased substantially the relative risk ratio between malnutrition and severe sarcopenia (RRR = 11.23 versus 6.45). In general, factors associated with sarcopenia status were similar irrespective of the cut-off point adopted. Higher cut-off points for the definition of weakness may be better for identifying sarcopenia, enabling early interventions to avoid adverse outcomes related to the disease.

## Resumo

O objetivo foi comparar a prevalência de sarcopenia e os fatores associados à definição de fraqueza muscular estabelecida com dois diferentes pontos de corte para a força de preensão palmar. Foi realizado um estudo transversal com 7.065 participantes (com 50 anos ou mais) do Estudo ELSI-Brasil. A sarcopenia foi definida pelo *European Working Group on Sarcopenia en Older People* (EWGSOP2), e dois pontos de corte para a força de preensão palmar para definir a fraqueza: < 27kg para homens/< 16kg para mulheres ou < 36kg para homens/< 23kg para mulheres. A prevalência dos diferentes estados de sarcopenia foi estimada, e as associações com fatores sociodemográficos, comportamentais e clínicos foram investigadas por meio de modelos de regressão multinomial. A idade média dos participantes foi de 61 anos; 51,8% eram mulheres e 41,5% estavam em risco de desnutrição. Ao adotar os pontos de corte mais elevados (< 36kg/< 23kg) para a definição de fraqueza, a prevalência de sarcopenia provável e de sarcopenia quadruplicou (40,1% *versu*s 10,6%; 5% *versus* 1,4%, respectivamente) e a prevalência de sarcopenia grave dobrou (8,8% *versus* 3,9%). O estado nutricional foi associado ao *status* de sarcopenia; entretanto, os pontos de corte < 36kg/< 23kg aumentaram substancialmente a razão de risco relativo entre desnutrição e sarcopenia grave (RRR = 11,23 *versus* 6,45). De forma geral, os fatores associados aos estados de sarcopenia foram semelhantes, independentemente do ponto de corte adotado. Pontos de corte mais elevados para a definição de fraqueza podem ser mais adequados para a identificação da sarcopenia, permitindo intervenções precoces para evitar desfechos adversos relacionados à doença.

## Resumen

El objetivo de este estudio fue comparar la prevalencia de sarcopenia y sus factores asociados con la definición de debilidad muscular establecida con dos puntos de corte diferentes para la fuerza de agarre. Se realizó un estudio transversal con 7.065 participantes (de 50 años o más) de la Encuesta ELSI-Brazil. La sarcopenia fue definida por el *European Working Group on Sarcopenia en Older People* (EWGSOP2), y dos puntos de corte para la fuerza de agarre para definir la debilidad: < 27kg para hombres/< 16kg para mujeres o < 36kg para hombres/< 23kg para mujeres. Se estimó el predominio de diferentes estados de sarcopenia, y se examinaron las asociaciones con factores sociodemográficos, conductuales y clínicos a partir de modelos de regresión multinomial. La edad media de los participantes fue de 61 años; el 51,8% eran mujeres; y el 41,5% estaban en riesgo de desnutrición. Al adoptar los puntos de corte más altos (< 36kg/< 23kg) para determinar la debilidad, el predominio de una posible sarcopenia y de sarcopenia se cuadruplicó (40,1% frente a 10,6%; 5% frente a 1,4%, respectivamente) y el predominio de sarcopenia grave se duplicó (8,8% frente a 3,9%). El estado nutricional se asoció con estados de sarcopenia; sin embargo, los puntos de corte < 36kg/< 23kg aumentaron sustancialmente la relación de riesgo relativo (RRR) entre la desnutrición y la sarcopenia grave (RRR = 11,23 frente a 6,45). En general, los factores asociados con estados de sarcopenia fueron similares, independientemente del punto de corte adoptado. Los puntos de corte más altos para determinar la debilidad pueden ser más apropiados en la identificación de la sarcopenia, lo que permite intervenciones tempranas con el fin de evitar resultados adversos relacionados con la enfermedad.

## Introduction

Sarcopenia is a generalised disease of skeletal muscle characterised by the concurrent combination of reduced muscle mass and muscle strength ^1^ ^,^ ^2^. It is associated with an increased risk of falls, physical dependence, and death ^3^. Given the different definitions and forms of assessing sarcopenia based on muscle strength and mass present in the literature, considerable variability is found in the prevalence of the disease. According to a meta-analysis conducted by Petermann-Rocha et al. ^4^, the global prevalence of sarcopenia ranges from 10% to 27% among individuals older than 60 years of age when defined by the International Working Group on Sarcopenia (IWGS) ^5^, the European Working Group on Sarcopenia in Older People (EWGSOP ^6^ and EWGSOP2 ^3^), the Foundation for the National Institutes of Health Biomarkers Consortium Sarcopenia Project (FNIH) ^7^, the Asian Working Group for Sarcopenia (AWGS) ^8^, or low muscle mass ^9^ ^,^ ^10^. When defined by the EWGSOP 2, the prevalence of probable sarcopenia ranges from 0.8 to 73% and the prevalence of severe sarcopenia ranges from 0% to 3.8% ^11^ ^,^ ^12^ ^,^ ^13^ ^,^ ^14^ ^,^ ^15^. In the Brazilian population, the prevalence of sarcopenia ranges from 4.8% to 62% ^16^ ^,^ ^17^, and according to the EWGOSP2, the prevalence of probable sarcopenia ranges from 10.1% to 50% ^18^ ^,^ ^19^ ^,^ ^20^ and the prevalence of severe sarcopenia ranges from 2.5% to 3.2% ^18^ ^,^ ^19^. 

Recently, two operationalizations for sarcopenia were proposed. In 2020, the Sarcopenia Definition and Outcomes Consortium suggested that the definition of sarcopenia must include both weakness, defined by low grip strength, and slowness, defined by low usual gait speed ^21^. The other operationalization is proposed by the EWGSOP ^3^, which updated version (EWGSOP2, 2019) classifies low muscle strength as probable sarcopenia and low strength combined with low muscle mass as sarcopenia. The addition of low physical performance defines severe sarcopenia ^3^. Despite the absence of a consensus on how to assess and diagnose sarcopenia, the operationalization by the EWGSOP2 still considers the assessment of muscle mass in the diagnosis and is the only one endorsed by a range of international scientific societies for clinical practice and research ^22^. 

In the first version of the EWGSOP ^6^, muscle mass was the main parameter for the diagnostic criteria of sarcopenia. However, scientific evidence accumulated up to the current version of the consensus (EWGSOP2) ^3^ points to muscle strength as the best indicator of adverse health events ^23^ ^,^ ^24^. Thus, muscle strength became the primary parameter for the diagnosis of sarcopenia, exerting an impact on the prevalence of the disease and its association with negative outcomes in older adults ^25^ ^,^ ^26^.

The EWGSOP2 recommends cut-off points for grip strength of < 27kg for men and < 16kg for women, established based on 2.5 standard deviations below the normative average in Great Britain ^27^. However, various cut-off points are found in the literature, ranging from 16kg to 31kg for women and 26kg to 40kg for men ^15^ ^,^ ^28^ ^,^ ^29^ ^,^ ^30^ ^,^ ^31^ ^,^ ^32^ ^,^ ^33^ ^,^ ^34^ ^,^ ^35^. These cut-off points were defined based on negative outcomes, which makes them clinically relevant. Among these cut-off points, the ones by Spexoto et al. ^15^ were the only ones defined through receiver operating characteristic curves analysis that considered death as outcome, using a large English sample (≥ 60 years of age) in 14-year follow-up. Those cut-off points were < 36kg for men/< 23kg for women and were the only cut-off points among all those analysed capable of identifying the risk of mortality for all sarcopenia status defined by the EWGSOP2.

It is important to consider that as the grip strength cut-off points < 36kg for men and < 23kg for women were based on the outcome death, for such outcome, tests with high sensitivity are recommended (diagnosing most patients even at the expense of a high percentage of false positives), as the purpose of the test is to rule out this outcome ^36^. It is observed in the study by Spexoto et al. ^15^ that high sensitivity was prioritized for determining the highest cut-off points, as indicated by the sensitivity values of 58.6% for grip strength < 36kg for men and sensitivity of 68.9% for grip strength < 23kg for women, while for the cut-off points proposed by the EWGSOP2 it was found for grip strength < 27kg for men a sensitivity of 18.5% and for grip strength < 16kg for women a sensitivity of 23.6%, considering death as outcome.

The systematic review by Fernandes et al. ^37^ has already showed the impact on sarcopenia prevalence when grip strength cut-off points different from those established by the consensus are used, but to date, no comparisons have been performed for the prevalence of sarcopenia and associated factors when different cut-off points are used to define muscle weakness in a Brazilian sample. Therefore, we tested the following hypotheses: (i) the use of higher cut-off points for grip strength to define weakness considerably increases the prevalence of the disease and (ii) factors associated with sarcopenia are the same irrespective of the cut-off point adopted to define weakness. The confirmation of these hypotheses may suggest that adopting higher cut-off points for grip strength to define muscle weakness would enable early diagnosis and treatment of the disease.

## Methods

### Study population

We analysed data from the first wave of the *Brazilian Longitudinal Study of Aging* (ELSI-Brazil, acronym in Portuguese). ELSI-Brazil is a home based survey, conducted in a nationally representative sample of Brazilians aged 50 years and over ^38^. The baseline survey (wave 1), conducted in 2015-2016 was composed of 9,412 individuals, 2,347 of whom were excluded from the present cross-sectional analysis due to missing data on the variables of interest and outcome. The final analysed sample was composed of 7,065 individuals aged 50 or older. Further information on the ELSI-Brazil Study can be found in a previous publication ^38^ and in the study homepage (https://elsi.cpqrr.fiocruz.br/en/home-english/).

### Sarcopenia

Sarcopenia was defined according to the EWGSOP2 ^3^, which involves the assessment of strength, muscle mass and physical performance.

Muscle strength was measured using an adjustable handgrip dynamometer (SAEHAN, Sh5002, http://www.saehanmedical.com/) ^39^. During the test, the participant remained sitting in an armless chair, arm alongside the trunk, elbow flexed at 90º, forearm in neutral position and thumb pointing upward. Three maximum grip strength trials were performed with the dominant hand and a one-minute rest interval between trials. The highest value was considered for analysis. We compared two cut-off points for the definition of muscle weakness: < 27kg for men/< 16kg for women based on the EWGSOP2 ^3^ and < 36kg for men/< 23kg for women based on Spexoto et al. ^15^, the latter of which was associated with a greater risk of mortality for all sarcopenia status.

Skeletal muscle mass (SMM) was estimated using the equation proposed by Lee ^40^, which considers weight, height, sex, age and ethnicity for the calculation. This equation was validated for Brazilian older adults considering DXA (dual x-ray absorptiometry) as the gold standard; no statistically significant difference was found in the estimate of SMM between the two methods ^41^. Estimated SMM was adjusted by height squared (SMM/height^2^) to determine the skeletal muscle mass index (SMMI) ^9^. Low muscle mass was defined by the 20th percentile of the population distribution and was 7.07kg/m^2^ for women and 9.54kg/m^2^ for men ^42^.

Physical performance was assessed using the three-meter walk test performed twice with a one-minute rest interval between trials. The participant was instructed to walk at his/her habitual pace to a distance marked on the floor. Gait speed was determined by the distance walked in meters divided by the shorter time performed by the participants in seconds (expressed as m/s). Gait speed ≤ 0.8 m/s indicated low physical performance ^3^. 

The EWGSOP2 establishes four sarcopenia status. The first category is the non-sarcopenic group, defined by individuals without low muscle strength; this is the reference category. The second category is probable sarcopenia, which is defined by the presence of only low muscle strength. The third category is sarcopenia, which is recorded for individuals with low muscle strength and low muscle mass, but the physical performance is good. And the fourth and last category is severe sarcopenia, which is recorded for individuals with low muscle strength, low muscle mass and low physical performance. The sarcopenia status in our study was defined in accordance with this classification ^3^.

### Variables of interest

The variables of interest analysed cover a wide range of factors associated with sarcopenia in the literature. The socioeconomic variables were sex, age in years, marital status (with/without a conjugal life), schooling (illiterate; 1-4; 5-8; and ≥ 9 years of study) ^43^, household income (in quintiles) ^44^ and skin colour (white, black/brown or Asian descendant).

The behavioural variables were smoking, alcohol intake and physical activity level. Individuals were classified as non-smokers, ex-smokers, and smokers. The smoking burden was calculated for the last two categories by the number of pack-years (number of cigarettes smoked per day times number of years smoking divided by 20) ^44^. Alcohol intake was categorized based on frequency: rarely or never (up to once per week), frequently (two to six days per week) or daily ^43^. Physical activity level was determined using the Brazilian version of the *International Physical Activity Questionnaire* (IPAQ) ^45^. The participants were classified based on the recommendations of the World Health Organization: “insufficiently active” (moderate physical activity < 150 minutes and vigorous physical activity < 75 minutes per week); “active” (moderate physical activity ≥ 150 minutes and vigorous physical activity ≥ 75 minutes per week or a combination of both types of activity ≥ 150 minutes per week) ^46^.

Clinical conditions were recorded based on self-reports of a medical diagnosis of cancer, heart failure, infarction, stroke, lung disease, osteoporosis, spine osteoarthritis and diabetes. For the latter, duration in years was also analysed. Arterial hypertension was defined as systolic blood pressure ≥ 140mmHg or diastolic blood pressure ≥ 90mmHg, irrespective of the self-report of a medical diagnosis ^47^. Memory was assessed based on the global immediate and delayed recall score on the word list test (score: 0 to 20 words) ^48^. 

Nutritional status was assessed using the adapted Mini Nutritional Assessment (MNA) screening measure, which addresses six aspects: change in food intake, weight loss in previous months, mobility, psychological stress, or acute disease in previous three months, neuropsychological problems, and body mass index (BMI). The score ranges from 0 to 14 points and is used to classify nutritional status as normal (12 to 14 points), at risk of malnutrition (8 to 11 points) and malnutrition (0 to 7 points) ^49^ ^,^ ^50^.

### Statistical analysis

The characteristics of the sample were expressed as means and standard deviations for quantitative variables and proportions for qualitative variables. The prevalence of sarcopenia was defined with a 95% confidence interval (95%CI) using the two cut-off points for grip strength to define weakness. Multinomial regression was performed to determine factors associated with the states of sarcopenia. Associations with a p-value < 0.2 in the bivariate analysis were incorporated into the multiple regression model using the stepwise method. In the adjusted analysis, a p-value < 0.05 was considered indicative of statistical significance. As the ELSI-Brazil Study has a complex sampling design, the analyses were performed with sampling weight, which is essential for population inferences. The Stata 16.1 SE program (https://www.stata.com) was used for all statistical analyses. 

### Ethical approval and informed consent

The ELSI-Brazil Study complies with all ethical precepts required for scientific studies carried out with human beings, such as voluntary participation, informed consent, privacy of participants and confidentiality of information. The ELSI-Brazil Study received approval from the Human Research Ethics Committee of the Oswaldo Cruz Foundation − Minas Gerais (approval certificate number: 34649814.3.0000.5091). All participants signed a statement of informed consent.

## Results

The mean age of the participants was 61 years. The sample was composed predominantly of women (51.8%) and individuals with a conjugal life (67%). The most prevalent health conditions were spine osteoarthritis (41%), hypertension (38.2%) and osteoporosis (15.1%). Based on the MNA screening, 41.5% of the sample were at risk of malnutrition and 10% were classified as malnourished. With regards to behavioural characteristics, 47.6% reported being non-smokers, 87.1% reported rare alcohol intake and 53.8% had a sedentary lifestyle. The characteristics of the participants are displayed in Tables 1 and 2.

**Table 1:** Sociodemographic and behavioral characteristics of participants of the *Brazilian Longitudinal Study of Aging* (ELSI-Brazil), 2015-2016 (N = 7,065).

Variables	Total
Sociodemographic characteristics	
Age [years] (mean ± SD)	61.7 ± 9.1
Age group [years] (%)	
50-59	50.1
60-69	30.0
70-79	14.8
≥ 80	5.1
Sex [female] (%)	51.8
Marital status [without conjugal life] (%)	33.0
Skin color	
White	44.3
Black/Brown	54.6
Asian descendant	1.1
Household income [quintile] (%)	
1^st^ (highest)	21.6
2^nd^	21.1
3^rd^	19.7
4^th^	19.3
5^th^ (lowest)	18.3
Schooling [years] (%)	
≥ 9	29.3
5-8	22.9
1-4	37.0
Illiterate	10.8
Behavioral characteristics	
Smoking (%)	
Non-smoker	47.6
Ex-smoker	35.3
Smoker	17.1
Smoking burden [pack-years] (mean ± SD)	25.9 ± 28.4
Alcohol intake (%)	
Rarely or never	87.1
Frequently	9.4
Daily	3.5
Sedentary lifestyle (%)	53.8

SD: standard deviation.

Source: data from the first wave of the ELSI-Brazil Study, 2015-2016.

Note: data are shown as proportions, means, and standard deviations calculated considering sample weight.

**Table 2:** Clinical and anthropometric characteristics of participants of the *Brazilian Longitudinal Study of Aging* (ELSI-Brazil), 2015-2016 (N = 7,065).

Variables	Total
Clinical characteristic	
Arterial hypertension [yes] (%)	38.2
Diabetes [yes] (%)	14.6
Duration of diabetes [years] (mean ± SD)	9.4 ± 10.0
Cancer [yes] (%)	5.2
Lung disease [yes] (%)	8.5
Heart failure [yes] (%)	6.7
Infarction [yes] (%)	5.3
Stroke [yes] (%)	4.2
Spine osteoarthritis [yes] (%)	41.0
Osteoporosis [yes] (%)	15.1
Memory, score (mean ± SD)	7.4 ± 3.2
MNA - screening (%)	
Normal nutritional status [12-14]	48.5
Risk of malnutrition [8-11]	41.5
Malnutrition [≤ 7]	10.0
Components of sarcopenia	
Grip strength [kg] (mean ± SD)	
Men	35.5 ± 8.9
Women	21.5 ± 6.0
Skeletal muscle mass index [kg/m^2^] (mean ± SD)	
Men	10.6 ± 1.2
Women	8.4 ± 1.5
Gait speed [m/s] (mean ± SD)	0.8 ± 0.3
Prevalence of sarcopenia [< 27kg for men/< 16kg for women] (%)	
Non-sarcopenic	84.1
Probable sarcopenia	10.6
Sarcopenia	1.4
Severe sarcopenia	3.9
Prevalence of sarcopenia [< 36kg for men/< 23kg for women] (%)	
Non-sarcopenic	46.1
Probable sarcopenia	40.1
Sarcopenia	5.0
Severe sarcopenia	8.8

SD: standard deviation; MNA: Mini Nutritional Assessment.

Source: data from the first wave of the ELSI-Brazil Study, 2015-2016.

Note: data are shown as proportions, means, and standard deviations calculated considering sample weight.

**Figure 1:**
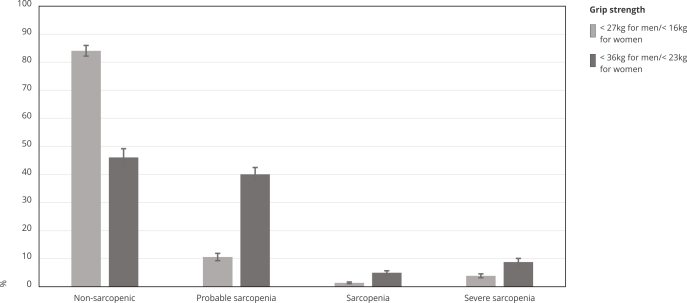
Prevalence (%) and 95% confidence intervals of sarcopenia according to cut-off points for grip strength to define weakness of < 27kg for men/< 16kg for women and < 36kg for men/< 23kg for women. *Brazilian Longitudinal Study of Aging* (ELSI-Brazil), 2015-2016.

Source: data from the first wave of the ELSI-Brazil Study, 2015-2016. Note: prevalence was calculated considering sample weight.

The prevalence proportion of probable sarcopenia, sarcopenia and severe sarcopenia (Figure 1) were lower when the cut-off point to define low muscle strength was < 27kg for men/16kg for women than when < 36kg for men/23kg for women (10.6%, 95%CI: 9.3-12.0 versus 40.1%, 95%CI: 37.7-42.5; 1.4%, 95%CI: 1.1-1.8 versus 5%, 95%CI: 4.3-5.8; 3.9%, 95%CI: 3.2-4.9 versus 8.8%, 95%CI: 7.5-10.3, respectively). The rates of sarcopenia according to the cut-off points for grip strength of 27kg for men/16kg for women and 36kg for men/23kg for women, per age category are displayed in Table 3.

**Table 3:** Prevalence (%) and 95% confidence interval (95%CI) of sarcopenia according to cut-off points for grip strength to define weakness of < 27kg for men/< 16kg for women) and < 36kg for men/< 23kg for women per age group in Brazil. *Brazilian Longitudinal Study of Aging* (ELSI-Brazil), 2015-2016 (N = 7,065).

Age group (years)	Prevelance of sarcopenia [% (95%CI)]							
	< 27kg for men/< 16kg for women				< 36kg for men/< 23kg for women			
	Non-sarcopenic	Probable sarcopenia	Sarcopenia	Severe sarcopenia	Non-sarcopenic	Probable sarcopenia	Sarcopenia	Severe sarcopenia
50-59	89.8 (87.9-91.4)	8.3 (6.9-9.9)	0.9 (0.6-1.4)	1.0 (0.7-1.6)	58.2 (55.0-61.4)	35.9 (32.9-39.0)	3.1 (2.5-3.9)	2.8 (2.1-3.7)
	[n = 2,871]	[n = 253]	[n = 26]	[n = 31]	[n = 1,845]	[n = 1,150]	[n = 100]	[n = 86]
60-69	86.3 (83.7-88.5)	9.8 (8.1-11.8)	1.2 (0.7-1.9)	2.7 (1.9-3.9)	42.9 (38.8-47.1)	43.3 (40.0-46.6)	5.6 (4.5-7.1)	8.2 (6.4-10.4)
	[n = 1,890]	[n = 228]	[n = 28]	[n = 64]	[n = 939]	[n = 982]	[n = 116]	[n = 173]
70-79	72.0 (68.2-75.5)	15.9 (13.4-18.8)	2.8 (1.9-4.3)	9.3 (7.5-11.4)	24.5 (21.1-28.4)	47.6 (44.0-51.1)	9.4 (7.4-11.7)	18.5 (15.9-21.4)
	[n = 915]	[n = 197]	[n = 34]	[n = 113]	[n = 304]	[n = 601]	[n = 116]	[n = 238]
80 or older	49.5 (43.7-55.5)	22.5 (16.8-29.3)	3.8 (2.2-6.3)	24.2 (19.2-30.0)	8.5 (5.8-12.2)	40.6 (33.7-47.7)	6.8 (4.9-9.4)	44.1 (38.0-50.6)
	[n = 211]	[n = 85]	[n = 16]	[n = 103]	[n = 35]	[n = 165]	[n = 29]	[n = 186]

Source: data from the first wave of the ELSI-Brazil Study, 2015-2016.

Note: prevalences were calculated considering sample weight.

Table 4 displays the estimated parameters of the multinomial regression models for sarcopenia status with two cut-off points of grip strength to define weakness. The increase in age was the only variable associated with all sarcopenia status irrespective of the cut-off point adopted.

**Table 4:** Factors associated with different sarcopenia status among participants of the *Brazilian Longitudinal Study of Aging* (ELSI-Brazil), 2015-2016 (n = 7,065).

	Adjusted model [RRR (95%CI)]					
	Probable sarcopenia		Sarcopenia		Severe sarcopenia	
	Sarcopenia 27kg/16kg	Sarcopenia 36kg/23kg	Sarcopenia 27kg/16kg	Sarcopenia 36kg/23kg	Sarcopenia 27kg/16kg	Sarcopenia 36kg/23kg
Age [years]	1.03 (1.02-1.04) *	1.05 (1.04-1.06) *	1.08 (1.05-1.10) *	1.10 (1.08-1.12) *	1.13 (1.11-1.15) *	1.17 (1.15-1.19) *
Female [sex]	0.95 (0.76-1.20)	1.04 (0.87-1.24)	0.42 (0.24-0.73) *	0.53 (0.38-0.75) *	0.75 (0.47-1.19)	1.03 (0.79-1.36)
Without conjugal life	1.02 (0.81-1.28)	1.10 (0.94-1.30)	1.04 (0.69-1.56)	1.39 (0.98-1.95)	1.05 (0.74-1.49)	1.34 (1.09-1.64) *
Income [quintile]						
2^nd^	1.25 (0.91-1.72)	1.03 (0.80-1.33)	0.65 (0.23-1.84)	0.72 (0.45-1.14)	1.39 (0.72-2.70)	1.12 (0.73-1.72)
3^rd^	1.21 (0.87-1.67)	1.09 (0.86-1.38)	1.70 (0.72-4.02)	1.27 (0.81-1.97)	1.75 (0.97-3.15)	1.38 (0.93-2.04)
4^th^	1.19 (0.86-1.65)	1.25 (0.97-1.61)	1.52 (0.65-3.59)	1.32 (0.83-2.11)	1.77 (0.95-3.28)	1.71 (1.10-2.65) *
5^th^ (lowest)	1.49 (1.10-2.02) *	1.41 (1.08-1.83) *	1.19 (0.49-2.85)	1.47 (0.98-2.21)	1.95 (1.05-3.61) *	1.78 (1.22-2.59) *
Smoking burden [pack-years]	1.00 (0.99-1.00)	1.00 (1.00-1.00)	1.00 (0.99-1.01)	1.00 (0.99-1.00)	1.00 (1.00-1.01)	1.00 (1.00-1.01)
Alcohol intake						
Frequently	0.71 (0.44-1.14)	0.83 (0.65-1.07)	1.11 (0.37-3.40)	0.78 (0.47-1.29)	1.05 (0.49-2.25)	0.76 (0.50-1.17)
Daily	0.46 (0.18-1.17)	0.61 (0.38-0.96) *	1.27 (0.37-4.39)	1.06 (0.57-1.98)	0.60 (0.28-1.27)	0.69 (0.42-1.14)
Sedentary lifestyle [yes]	1.44 (1.18-1.77) *	1.25 (1.07-1.47) *	1.54 (0.91-2.61)	1.21 (0.93-1.58)	1.18 (0.78-1.76)	0.92 (0.69-1.23)
Time since the diagnosis of diabetes [years]	1.02 (1.01-1.04) *	1.03 (1.02-1.05) *	0.98 (0.94-1.02)	0.97 (0.92-1.03)	0.99 (0.96-1.02)	1.01 (0.98-1.03)
Cancer [yes]	1.02 (0.65-1.60)	1.26 (0.96-1.65)	1.72 (0.79-3.74)	1.85 (1.13-3.04) *	0.71 (0.40-1.27)	0.92 (0.61-1.40)
Lung disease [yes]	1.74 (1.23-2.46) *	1.21 (0.99-1.47)	1.22 (0.46-3.26)	1.16 (0.69-1.96)	1.34 (0.82-2.19)	1.04 (0.67-1.63)
Heart failure [yes]	1.34 (0.98-1.83)	1.23 (0.88-1.72)	1.48 (0.55-3.97)	0.71 (0.35-1.46)	0.78 (0.50-1.21)	0.89 (0.55-1.46)
Infarction [yes]	0.91 (0.66-1.25)	1.00 (0.76-1.30)	0.61 (0.26-1.43)	0.94 (0.47-1.88)	0.62 (0.28-1.39)	1.10 (0.57-2.13)
Stroke [yes]	3.12 (2.05-4.75) *	2.31 (1.55-3.43) *	0.72 (0.15-3.51)	0.71 (0.28-1.75)	1.68 (1.04-2.70) *	1.46 (0.93-2.27)
Spine osteoarthritis [yes]	1.28 (1.04-1.58) *	1.16 (1.00-1.33) *	0.56 (0.35-0.92) *	0.80 (0.59-1.08)	0.81 (0.58-1.14)	0.81 (0.61-1.09)
Osteoporosis [yes]	1.41 (1.12-1.77) *	1.27 (1.05-1.53) *	1.38 (0.68-2.81)	1.18 (0.80-1.73)	1.37 (0.88-2.12)	1.21 (0.88-1.66)
Memory [score]	0.92 (0.89-0.94) *	0.93 (0.91-0.95) *	1.04 (0.95-1.15)	1.00 (0.94-1.06)	0.86 (0.81-0.91) *	0.92 (0.88-0.97) *
MNA - screening						
Risk of malnutrition (8-11)	1.14 (0.90-1.44)	1.19 (1.04-1.35) *	2.96 (1.58-5.53) *	3.31 (2.45-4.46) *	1.80 (1.23-2.64) *	2.45 (1.89-3.17) *
Malnutrition (≤ 7)	0.91 (0.64-1.30)	1.21 (0.93-1.57)	4.96 (1.95-12.58) *	6.19 (3.91-9.81) *	6.45 (4.18-9.94) *	11.23 (7.90-15.96) *

95%CI: 95% confidence interval; MNA: Mini Nutritional Assessment; RRR: relative risk ratio.

Source: data from the first wave of the ELSI-Brazil Study, 2015-2016.

Note: the reference category for sarcopenia status was the non-sarcopenic group, which is omitted in this table. Models adjusted for age, sex, marital status, total household income, smoking burden (pack-years), alcohol intake, physical activity level, duration of diabetes (years), cancer, lung disease, heart failure, infarction, stroke, spine osteoarthritis, osteoporosis, memory performance and MNA screening. Muscle mass is based on the 20th percentile of the population. Sarcopenia 27kg/16kg - low muscle strength < 27kg/16kg; Sarcopenia 36kg/23kg − low muscle strength < 36kg/23kg.

* Statistical significance p < 0.05. Estimates were calculated considering sample weight.

In the final model, the following variables were associated with probable sarcopenia irrespective of the cut-off point adopted: low income, sedentary lifestyle, longer duration of diabetes, spine osteoarthritis, stroke, and osteoporosis; a better memory performance diminished the likelihood of having probable sarcopenia. Lung disease was associated with probable sarcopenia only when < 27kg/16kg was the cut-off point for grip strength. Daily alcohol intake and risk of malnutrition were associated with probable sarcopenia when < 36kg/23kg was the cut-off point for grip strength.

Irrespective of the cut-off point adopted, the risk of malnutrition and malnutrition were associated with a greater likelihood of sarcopenia, whereas the female sex diminished the likelihood of sarcopenia. Spine osteoarthritis was associated with a lower likelihood of sarcopenia when < 27kg/16kg was adopted as the cut-off point for grip strength. Cancer was associated with a greater likelihood of sarcopenia when < 36kg/23kg was adopted.

Irrespective of the cut-off point adopted, low income, risk of malnutrition and malnutrition were associated with severe sarcopenia, whereas a better memory performance diminished the likelihood of having severe sarcopenia. Stroke was associated with severe sarcopenia when < 27kg/16kg was adopted as the cut-off point for grip strength. The absence of a conjugal life was associated with severe sarcopenia when < 36kg/23kg was adopted as the cut-off point for grip strength.

## Discussion 

In the present study, we found that by adopting higher cut-off points for grip strength (< 36kg for men and < 23kg for women) to define weakness, the prevalence of probable sarcopenia and sarcopenia quadrupled, and the prevalence of severe sarcopenia doubled compared to the use of lower cut-off points (< 27kg for men and < 16kg for women). We also found that the factors associated with sarcopenia status were similar irrespective of the cut-off point used to define weakness.

Only the study conducted by Spexoto et al. ^15^, who investigated sarcopenia in a sample of 6,182 community-dwelling English people based on the EWGSOP2, compared the prevalence of the disease using cut-off points for grip strength of < 27kg/16kg versus < 36kg/23kg to define weakness. The authors described similar results to those of the present study: 9.6% prevalence of probable sarcopenia, 1.4% prevalence of sarcopenia and 3.8% prevalence of severe sarcopenia when the cut-off point for grip strength was < 27kg/16kg and 33.96% prevalence of probable sarcopenia, 6.2% prevalence of sarcopenia and 8.6% prevalence of severe sarcopenia when the cut-off point for grip strength was < 36kg/23kg. This seems to demonstrate that, independently of the population sample analysed, even with different socioeconomic, behavioural, and clinical characteristics, the use of lower or higher cut-off points for defining muscle weakness results in similar prevalence rates of the states of sarcopenia.

The literature reports that the prevalence of probable sarcopenia ranges from 0.8% to 73%, sarcopenia from 0% to 62% and severe sarcopenia from 0% to 3.2% ^11^ ^,^ ^12^ ^,^ ^13^ ^,^ ^14^ ^,^ ^16^ ^,^ ^17^ ^,^ ^18^ ^,^ ^19^. Such variability is the result of differences in the characteristics of the samples analysed, such as the inclusion of participants with higher mean age and non-representational samples, as well as different methods for defining each component of sarcopenia. Among the sarcopenia components, the muscle mass remains without consensus in the literature on the best technique for measuring it ^51^, hindering even more the prevalence identification, as the absence of a standard reference does not allow objective comparisons when different instruments are used. Moreover, although the EWGSOP2 algorithm is widely employed to diagnose sarcopenia, the uniting of the categories of the construct, such as uniting probable sarcopenia with non-sarcopenia, hinders the comparison of prevalence rates among studies ^20^ ^,^ ^26^ ^,^ ^52^ ^,^ ^53^. 

Besides finding higher rates of the sarcopenia status using the cut-off points for grip strength of < 36kg/23kg, which enables early identification and treatment of the disease, the way of obtaining these cut-off points was based on the outcome-guided approach rather than the biomarker-guided approach ^54^. The outcome-guided approach determines a cut-off point considering the association between an outcome and the biomarker, which is more recommended in the literature due to its clinical relevance ^36^ ^,^ ^54^. In contrast, the biomarker-guided approach divides the continuous measurement of this biomarker into percentages, mean or standard deviation. However, this approach can fail; as an exploratory method, it may not have good predictive capacity for adverse outcomes in older adults ^54^. With regards to using higher cut-off points for identifying weakness, studies have recently demonstrated that higher cut-off points than those proposed by the EWGSOP2 are associated with outcomes such as cardiometabolic multimorbidity ^55^ and mortality ^15^ ^,^ ^55^ ^,^ ^56^.

Proving our hypothesis, practically the same sociodemographic, behavioural, and clinical factors were associated with probable sarcopenia, sarcopenia, and severe sarcopenia irrespective of the cut-off point for grip strength adopted to define weakness. The sociodemographic factors common to the two cut-off points were an increase in age (associated with all states of sarcopenia), female sex (diminishing the likelihood of sarcopenia) and low income (increasing the likelihood of both probable sarcopenia and severe sarcopenia). These findings are consistent with data reported in previous studies found in the literature ^17^ ^,^ ^57^ ^,^ ^58^ ^,^ ^59^ ^,^ ^60^ ^,^ ^61^. Among behavioural factors, a sedentary lifestyle was associated with probable sarcopenia. It is widely known that low levels of physical activity result in low muscle strength and vice-versa at any age ^62^ ^,^ ^63^. 

Among the clinical factors common to both cut-off points, longer duration of diabetes, spine osteoarthritis, stroke and osteoporosis were associated with probable sarcopenia, whereas better memory performance was a protective factor for probable and severe sarcopenia. These conditions are known to exert an impact on musculoskeletal health as a consequence of endocrine, neuromuscular or cerebral changes that culminate in reductions in muscle strength, mass and function ^11^ ^,^ ^17^ ^,^ ^59^ ^,^ ^62^ ^,^ ^64^ ^,^ ^65^ ^,^ ^66^. The risk of malnutrition and malnutrition were also associated with sarcopenia and severe sarcopenia irrespective of the cut-off point adopted, confirming the importance of nutritional aspects among sarcopenic individuals profusely described in the literature ^17^ ^,^ ^58^. 

Most of the associations between factors and status of sarcopenia identified, irrespective of the cut-off point adopted for muscle weakness, had similar relative risk ratios (RRR). However, we found that the RRR were considerably higher for the risk of malnutrition and malnutrition when the cut-off point for grip strength was < 36kg/23kg, as an example, the RRR between malnutrition and severe sarcopenia practically doubled in comparison to the RRR of the same association when the cut-off point was < 27kg/16kg (RRR = 11.23 versus 6.45). It is alarming that more than half of the sample in the present study had nutritional problems (41.5% at risk of malnutrition and 10% malnourished). The greater risk of malnutrition and malnutrition associated with sarcopenia status may be explained by low protein and calorie intake by older adults, micronutrient deficiencies, malabsorption and age-related anorexia ^22^, which exert an impact on the synthesis of myofibrillary proteins ^67^, with the consequent loss of muscle mass and strength. Considering the effect size of the associations between nutritional status and sarcopenia status when < 36kg/23kg is used to define weakness and that the present sample has evident nutritional problems, the diagnosis of the different sarcopenia status using this cut-off point seems more adequate. 

Although the factors associated with sarcopenia status were generally similar when using the different cut-off points for grip strength to determine weakness, some were associated only when the higher cut-off points were adopted and others only when the lower cut-off points were adopted. These distinct associations may have been found because more individuals were diagnosed with sarcopenia status when the higher cut-off points were used. The addition of these individuals may have culminated in a statistically significant difference between groups (higher cut-off points) or annulled this difference (lower cut-off points).

The most likely interpretation of our study is that grip strength < 36kg/23kg is the best cut-off point for sarcopenia in the Brazilian population. We make this statement because the higher prevalence of the sarcopenia status at this cut-off point, especially for probable sarcopenia (40.1%), indicates the identification of a greater number of individuals who can receive early interventions, minimizing the progression to sarcopenia-related adverse health conditions, such as the occurrence of falls, physical disability and mortality ^3^. Furthermore, the factors associated with sarcopenia status were similar when the two cut-off points for grip strength were employed, indicating little difference concerning these factors when opting for the use of one cut-off point or the other.

Our study has several strong points, such as the use of a large representative national sample of the Brazilian population of community-dwelling older adults, encompassing an extensive gamut of sociodemographic and clinical characteristics of these participants. Another strong point is the use of simple, viable, validated measures for the assessment of sarcopenia, favouring the use of these measures in clinical practice to perform the diagnosis of the condition. Moreover, this was the first study to compare in a Brazilian sample the factors associated with sarcopenia status defined by the EWGSOP2 using two different cut-off points for muscle strength, enabling a direct analysis of the associations in the same study sample.

Some limitations of the study also need to be considered. First, the cross-sectional design does not enable establishing a temporal or cause-and-effect relationship between the associated factors and states of sarcopenia. Second, the data analysed were from community-dwelling individuals and therefore cannot be extrapolated to hospitalized individuals and those in assisted living facilities, among whom the prevalence of sarcopenia is expected to be higher. Lastly, the ELSI-Brazil Study does not objectively collect data on muscle mass using imaging equipment, which is the recommended method. However, muscle mass was estimated using an equation which does not invalidate our findings, as this equation has previously been validated using a gold standard method i.e., magnetic resonance ^40^.

## Conclusion

In this nationally representative sample of Brazilian community-dwelling older adults, the prevalence of both probable sarcopenia and sarcopenia was fourfold higher and the prevalence of severe sarcopenia was twofold higher when using higher cut-off points for grip strength (< 36kg for men/< 23kg for women) to define muscle weakness compared to the use of lower cut-off points (< 27kg for men/< 16kg for women). Sarcopenia status was significantly associated with sociodemographic, behavioural, and health-related factors and these factors were similar when the two cut-off points for weakness were employed. The use of a higher cut-off point for grip strength to define muscle weakness seems to be better for detecting sarcopenia in clinical practice, enabling early interventions to prevent adverse health outcomes related to this disease.
